# Intraoperative Trajectory Realignment Algorithm for Minimizing Screw Misplacement in Cervical Spinal Fusion

**DOI:** 10.34133/csbj.0222

**Published:** 2026-10-07

**Authors:** Pasit Komonsuwan, Jackrit Suthakorn, Shen Treratanakulchai, Sorayouth Chumnanvej

**Affiliations:** ^1^ Department of Biomedical Engineering, and Center for Biomedical and Robotics Technology (BART LAB), Faculty of Engineering, Mahidol University, Nakhon Pathom 73170, Thailand.; ^2^ Faculty of Medicine Ramathibodi Hospital, Mahidol University, Bangkok 10400, Thailand.

## Abstract

Spinal fusion is a surgical procedure designed to stabilize the spine by connecting vertebrae with screws and rods, where placement accuracy is critical, as even small deviations can cause severe neurovascular complications. The accuracy of traditional preoperative image-based navigation is limited by intraoperative alignment shifts, particularly in the highly mobile cervical spine. This study presents an intraoperative trajectory realignment algorithm that reconciles preoperative computed tomography data with simulated and phantom-derived intraoperative 2-dimensional (2D) projections by integrating forward kinematics, digitally reconstructed radiographs, and optimization-based search strategies. The algorithm was evaluated through computational simulations and physical experiments using a modular 3D-printed cervical spine phantom, with accuracy evaluated through clinical grading and assessment metrics, including entry/end point displacement, angular deviation, and minimum inter-trajectory distance. Results demonstrated high accuracy, with all screws graded as perfectly placed and deviations consistently below clinically relevant thresholds. While these findings confirm the feasibility of this approach under controlled experimental conditions, future validation using cadaveric models and real intraoperative fluoroscopic imaging remains necessary prior to clinical integration.

## Highlights

• Developed an intraoperative trajectory realignment framework integrating forward kinematics, digitally reconstructed radiographs, and optimization-based search to align preoperative CT with intraoperative imaging
• Demonstrated feasibility using 3D-printed cervical spine phantom experiments, achieving sub-millimeter alignment accuracy under controlled conditions
• Evaluated robustness across multiple intensity-based similarity metrics, showing consistent performance without substantial differences

## Graphical Abstract

**Figure:**
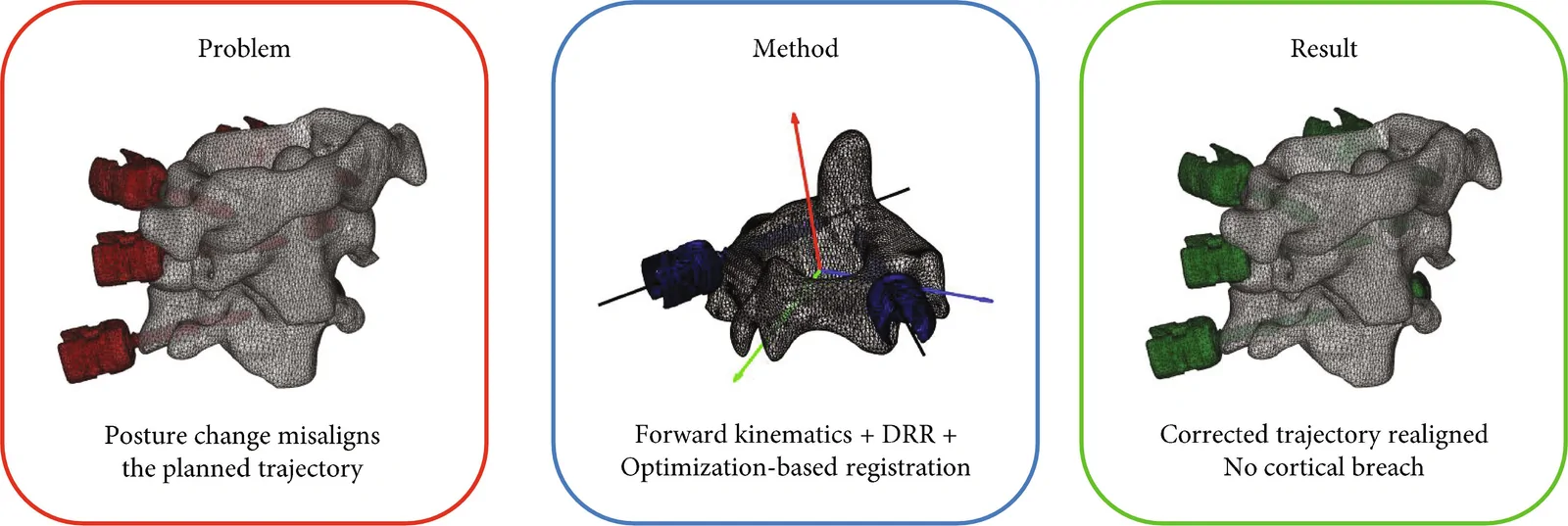

## Introduction

Cervical spinal fusion is a widely performed surgical procedure aimed at restoring stability and alleviating pain by rigidly connecting adjacent vertebrae. This is typically achieved through the insertion of pedicle or lateral mass screws, which are subsequently linked using rods or plates to maintain structural integrity [1–5].

Accurate screw placement is critical in cervical spine surgery due to the proximity of vital neurovascular structures. In conventional practice, screw insertion is primarily guided by the surgeon’s anatomical knowledge and intraoperative judgment [6–9]. However, even minor deviations from the intended trajectory may result in complications such as cortical breach, nerve root injury, or vascular damage [10,11]. These risks highlight the importance of precise trajectory planning and execution.

To improve surgical accuracy, a range of image-guided navigation systems and robotic-assisted techniques have been introduced. These systems integrate preoperative imaging with intraoperative guidance to enhance trajectory planning, reduce complication rates, and improve procedural efficiency [12–21]. Despite these advancements, most navigation frameworks rely heavily on preoperative imaging, assuming that the anatomical configuration remains consistent throughout the procedure.

In practice, this assumption is often violated. The cervical spine exhibits substantial mobility, and differences in patient positioning between preoperative imaging (typically acquired in the supine position) and intraoperative conditions (commonly in the prone position) can lead to substantial alignment discrepancies [5,22–24]. In addition, intraoperative factors such as surgical manipulation, muscle relaxation, and gravitational effects may further alter vertebral alignment. These factors introduce a mismatch between the planned trajectory and the actual anatomical configuration, potentially compromising the accuracy of navigation-guided screw placement [25,26].

Although current navigation systems incorporate registration techniques to mitigate these discrepancies, their ability to continuously compensate for intraoperative alignment changes remains limited. This gap motivates the need for methods that can dynamically update preoperative plans based on intraoperative imaging, thereby improving the robustness of navigation-assisted procedures.

A preliminary version of this work was previously presented in our conference paper [27], in which the core trajectory realignment algorithm and its initial computational validation were introduced. The present study extends that earlier work by incorporating phantom-based physical validation, additional comparative analysis of similarity metrics, computational efficiency assessment, and a more comprehensive discussion of methodological limitations and clinical translation.

In this study, an intraoperative trajectory realignment algorithm is proposed to address the discrepancy between preoperative planning and intraoperative spinal alignment. The method integrates forward kinematics, digitally reconstructed radiographs (DRRs), and optimization-based image registration to estimate the intraoperative pose of the cervical spine and update the planned screw trajectory accordingly. The approach is designed to complement existing navigation workflows by providing a mechanism for trajectory correction rather than replacing current systems.

The proposed algorithm is evaluated through both computational simulations and controlled physical experiments using a modular cervical spine phantom. While these experimental settings do not fully replicate the complexity of intraoperative fluoroscopic imaging, they provide a structured framework for assessing the feasibility and accuracy of the realignment strategy.

The remainder of this paper is organized as follows. The Cervical spine kinematics modeling section describes the modeling of cervical spine kinematics and reference frame construction using principal components analysis (PCA). The Simulated intraoperative images section presents the generation of simulated intraoperative images and alignment discrepancies. The Spine Image Registration and Trajectory Realignment section details the image registration and trajectory realignment algorithm. The Computational Experiment and Results section reports the computational evaluation, followed by the phantom-based validation in the Phantom Experiment and Results section. The Baseline Comparison section compares the proposed method with baseline similarity metrics, and the Computational Efficiency section analyzes computational performance. Finally, conclusions and future directions are discussed in the Conclusions section.

## Spine Kinematics

### Cervical spine kinematics modeling

The cervical spine is composed of 7 vertebrae (C1 to C7) that collectively enable a wide range of head and neck movements (Fig. 1). The upper cervical segment, consisting of the atlas (C1) and axis (C2), primarily facilitates nodding and axial rotation through specialized anatomical structures such as the odontoid process. In contrast, the subaxial vertebrae (C3 to C7) exhibit more uniform morphology and contribute to combined motions including flexion–extension, lateral bending, and rotation. The characteristic range of motion (ROM) at each cervical level provides important biomechanical constraints for modeling vertebral motion and is summarized in Table 1 [28–30].

**Fig. 1.**
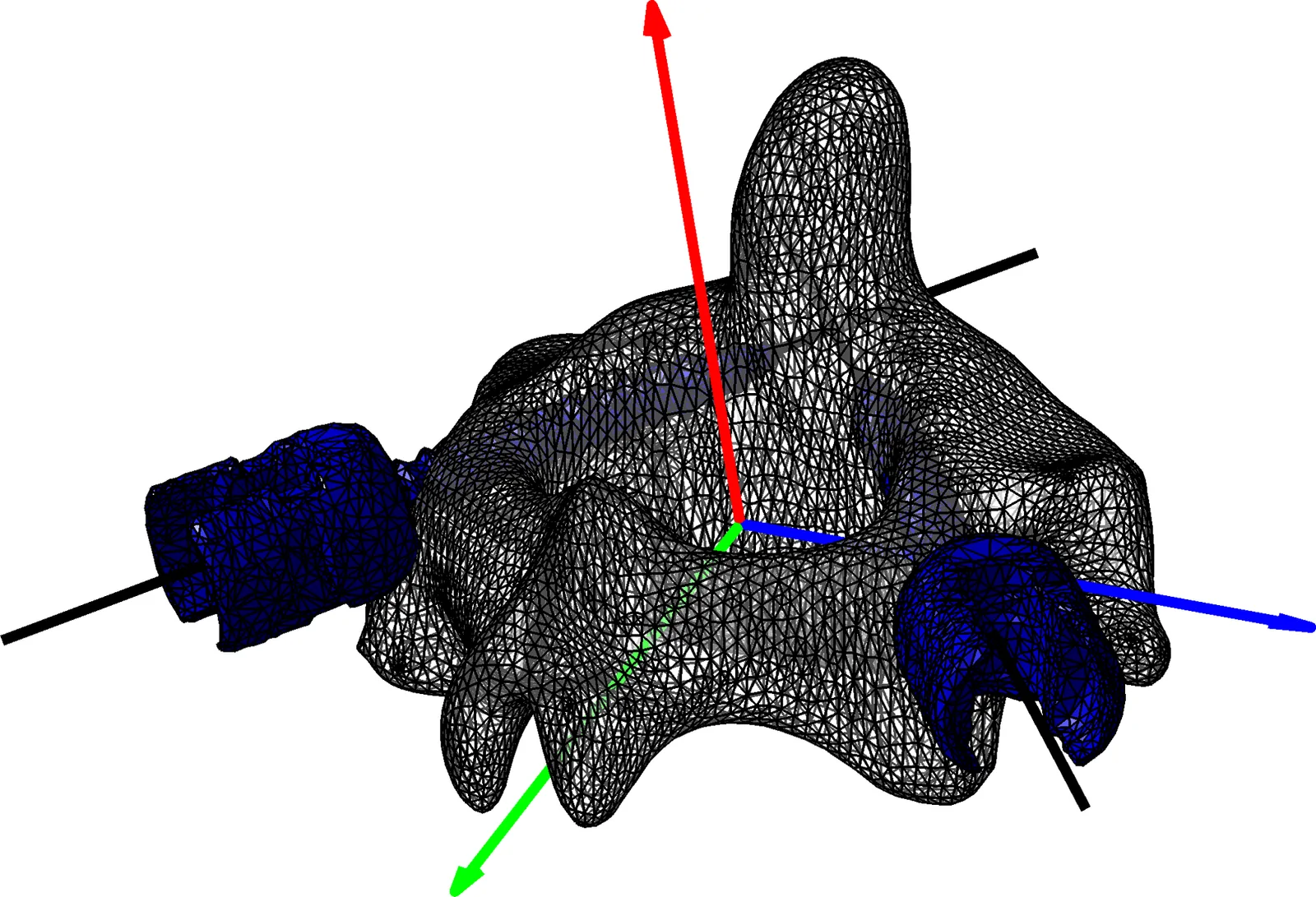
A cervical spine with reference frame constructed from PCA and its screw trajectory planning.

**Table 1.** Range of motion of cervical joints C1 to C7

Cervical joint	Flexion–extension	Rotation	Lateral bending
C0-C1	10–15°	5°	5°
C1-C2	20–25°	40–45°	5°
C2-C3	15–20°	5–10°	5°
C3-C7	15–20°	2–3°	10–15°

For computational modeling, patient-specific cervical spine geometries were obtained from the CTSpine1K dataset and reconstructed into 3-dimensional (3D) representations following segmentation of individual vertebrae [31,32]. These reconstructed models serve as the preoperative anatomical reference for trajectory planning and kinematic analysis.

To define the pose of each vertebra in a consistent and data-driven manner, a local coordinate system is established using PCA applied to the segmented 3D geometry [33]. The centroid of each vertebra is used as the origin of the local frame, while the principal axes derived from PCA define its orientation. This approach provides a reproducible method for estimating vertebral pose without requiring predefined anatomical landmarks, which may vary across patients.

Based on these local coordinate systems, the cervical spine is modeled as a serial kinematic chain in which adjacent vertebrae are connected through rigid transformations. Screw trajectories are initially defined in the preoperative configuration using 3D Slicer, following standard surgical planning practices [34–36]. The spatial relationship between vertebrae is then described using forward kinematics, allowing the propagation of transformations along the spine.

The pose of the *n*th vertebra can be expressed as:

$$C_{n}=\prod_{i=1}^{n}T_{i}\cdot C_{1}$$ (1)

where *C_n_* represents the rigid-body transformation [SE(3)] of the *n*th vertebra, parameterized by translation *p* = [*p_x_, p_y_, p_z_*] and rotation *R* = [*r_x_, r_y_, r_z_*] in XYZ-Euler angles. *T_i_* denotes the transformation between adjacent vertebrae in the kinematic chain. This formulation enables systematic modeling of cervical spine alignment and provides the mathematical foundation for subsequent image registration and trajectory realignment.

### Simulated intraoperative images

To estimate discrepancies between preoperative planning and intraoperative anatomy, a simulation framework was developed to generate imaging data under controlled alignment variations. This framework enables quantitative analysis of pose differences between the preoperative cervical spine model and its intraoperative configuration, which is essential for trajectory correction [37,38].

DRRs were employed to approximate intraoperative fluoroscopic images from the preoperative volumetric data. DRR generation involves projecting voxel-based 3D computed tomography (CT) data onto a 2D imaging plane by simulating x-ray attenuation along defined projection paths [39–41]. By manipulating the pose parameters of the cervical spine model, synthetic x-ray-like images corresponding to different spatial configurations can be produced.

Specifically, the DRR generation employed a parallel-ray orthographic projection model, computing pixel intensities via the direct linear summation of CT voxel values along the projection axis. Because this orthographic model simulates parallel rather than divergent rays, geometric magnification and fixed source-to-detector distances do not apply. To replicate the standard dual-view fluoroscopic workflow, the anteroposterior and lateral projections were generated independently by summing the volumetric data along orthogonal coordinate planes relative to the vertebral centroid.

Given the segmented vertebral models and their associated reference frames, the spine can be systematically transformed using the kinematic formulation described in the previous section. For each candidate pose, a corresponding DRR image is generated, providing a direct means of comparing projected images under different alignment conditions. This enables the formulation of an image-based registration problem, where the objective is to identify the pose parameters that best reconcile the simulated projections with intraoperative observations.

It should be noted that the DRR generation in this study assumes a simplified imaging model, including uniform attenuation properties and the absence of soft tissue, scatter, and noise effects. While this assumption facilitates controlled evaluation of the algorithm, it does not fully capture the complexity of real intraoperative fluoroscopy. The implications of this simplification are further discussed in the Conclusions section.

## Spine Image Registration and Trajectory Realignment

### Image registration

To estimate the intraoperative pose of the cervical spine, an image-based registration framework was formulated by comparing simulated projections with intraoperative imaging. The core idea is to iteratively adjust the pose of the preoperative model such that its projected appearance aligns with the observed intraoperative image. This registration framework builds upon our previously reported conference method [27], with further refinements and extended validation presented in the current study (Fig. 2).

**Fig. 2.**
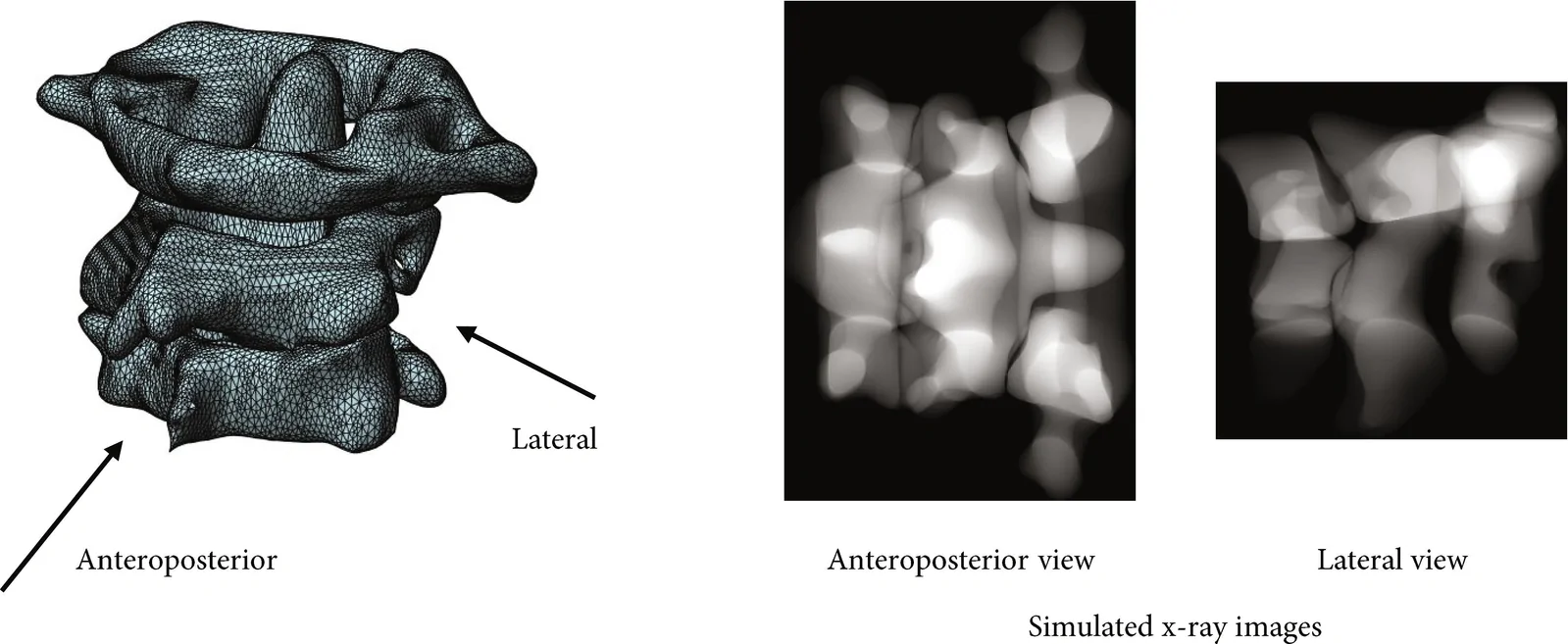
A voxel-based 3D imaging is used to replicate x-ray images with DRR method.

A difference image is first computed by subtracting the simulated projection from the reference intraoperative image. This residual image emphasizes mismatched regions, where higher intensity differences correspond to greater misalignment between the 2 images [42–44]. Minimizing this discrepancy provides a quantitative criterion for estimating the optimal pose parameters.

The registration problem is formulated as an optimization task in which the kinematic parameters of each vertebra are adjusted to minimize the difference between simulated and reference images. Each vertebra is parameterized by 6 degrees of freedom, comprising 3 translational components (*t_x_, t_y_, t_z_*) and 3 rotational components (*r_x_, r_y_, r_z_*). The similarity between images is quantified using the sum of absolute differences (SAD), defined as:

$$\mathrm{SAD}=\sum_{i=1}^{m}\sum_{j=1}^{n}\left|I_{\text{ref}}\left(i,\;j\right)-I_{\text{sim}}\left(i,\;j\right)\right|$$ (2)

where *I_ref_* denotes the intensity of the reference intraoperative image and *I_sim_* corresponds to the intensity of the simulated projection generated from the current pose estimate. Minimization of this cost function leads to alignment between the simulated and observed images [45,46] (Fig. 3).

**Fig. 3.**
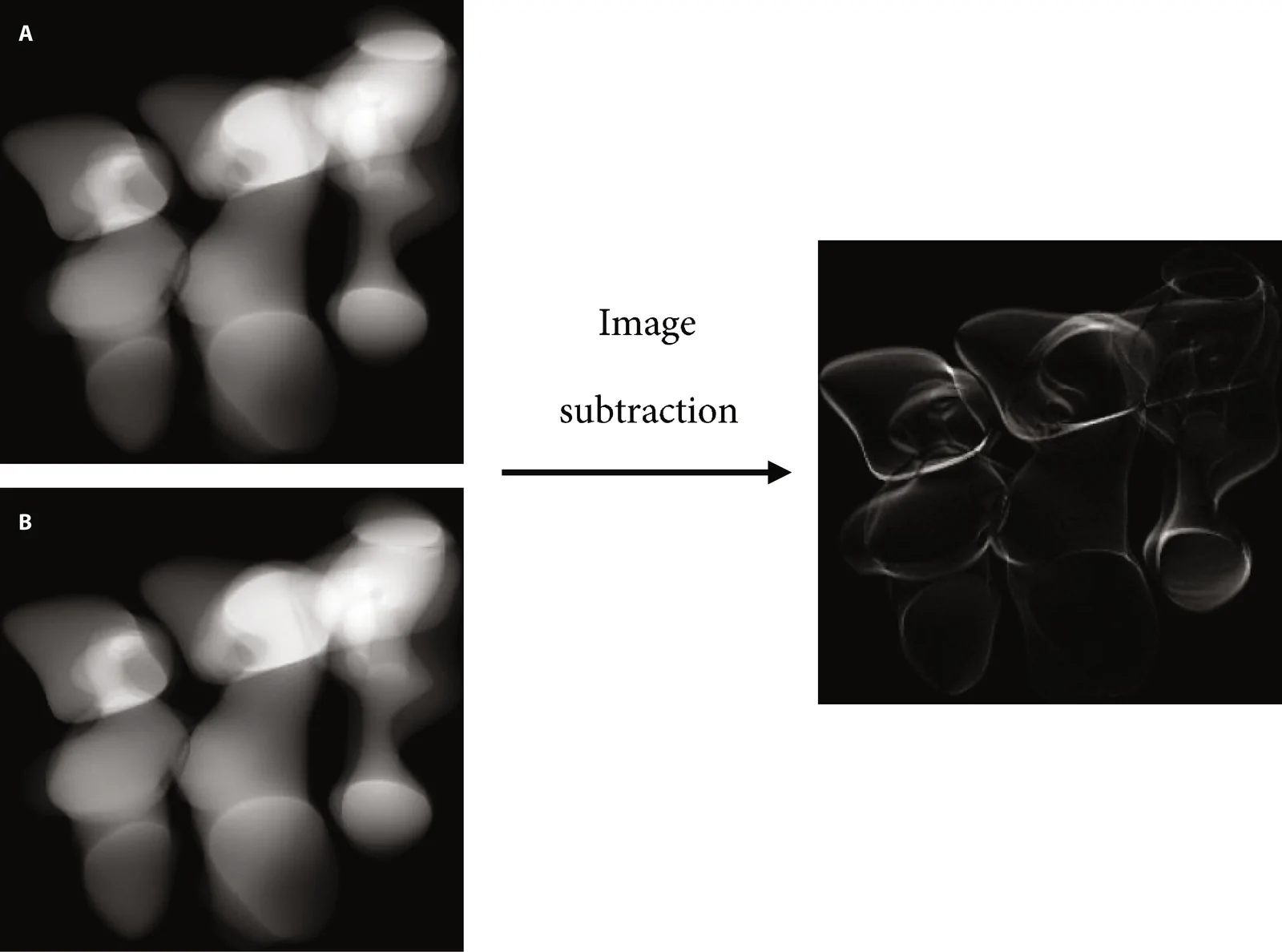
Difference image obtained from subtracting 2 DRR images generated under different pose parameters: (A) DRR generated using the reference pose parameters and (B) DRR generated using altered pose parameters. The resulting difference image highlights regions of spatial misalignment between the 2 projections.

The optimization was performed using a derivative-free pattern search algorithm implemented in MATLAB, which is suitable for nonconvex and nondifferentiable objective functions commonly encountered in image registration [47]. To improve computational tractability and avoid anatomically implausible solutions, the search space was constrained using physiological limits derived from the ROM of each cervical segment (Table 1) [30].

In this study, the registration process focused on the upper cervical vertebrae (C1 to C3), where fluoroscopic visibility is less affected by occlusion from surrounding anatomical structures such as the shoulders. The optimization was performed sequentially for each vertebral level, and the estimated transformations were propagated along the kinematic chain using forward kinematics [48,49]. This hierarchical approach enables consistent alignment across multiple vertebrae while maintaining biomechanical coherence.

The resulting optimized pose parameters define the intraoperative configuration of the cervical spine and serve as the basis for updating the preoperative screw trajectory [27,50,51]. An overview of the optimization workflow is illustrated in Fig. 4.

**Fig. 4.**
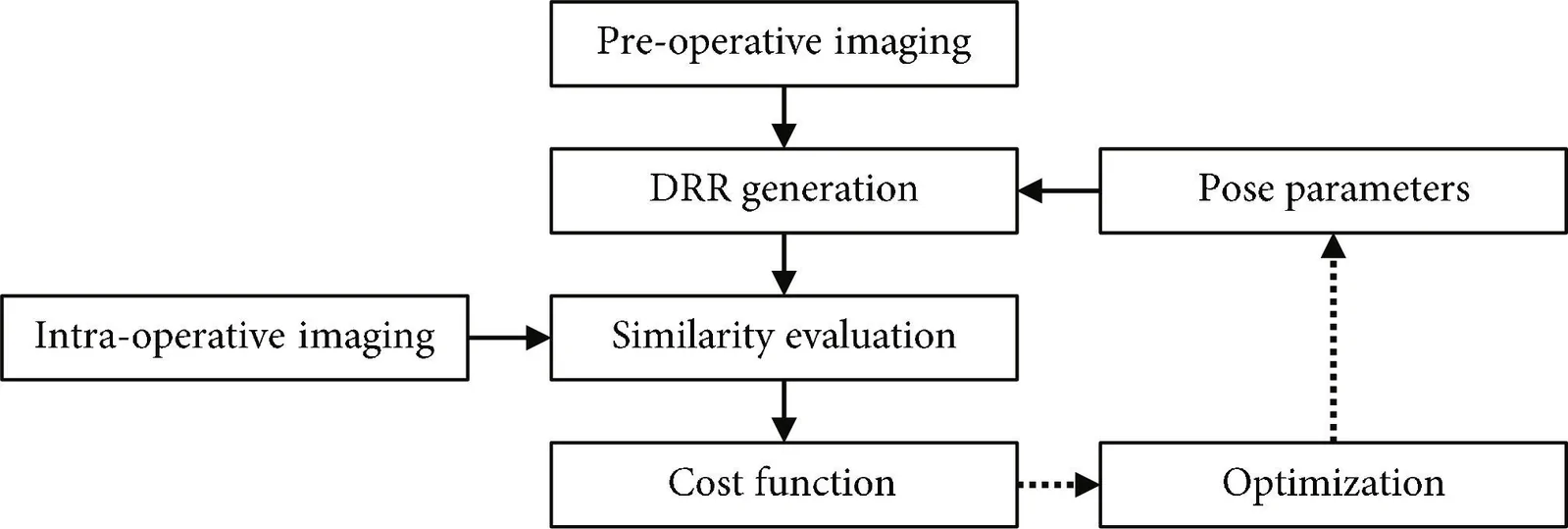
Overview of the optimization-based image registration framework for estimating intraoperative spine alignment.

### Trajectory realignment

Following the estimation of vertebral pose through image registration, the planned screw trajectory is updated to reflect the intraoperative configuration of the cervical spine. This update is performed by computing the relative transformation between the preoperative and estimated intraoperative coordinate frames.

Let *C* denote the preoperative reference frame of a vertebra and *C′* represent the corresponding intraoperative frame obtained from the optimization process. The transformation that maps the preoperative configuration to the intraoperative configuration is given by:

$$\text{∆}C=C^{-1}C^{\prime }$$ (3)

This relative transformation encodes both rotational and translational differences between the 2 states. The originally planned trajectory, defined in the preoperative frame as *t*, is then updated by applying this transformation:

$$t^{\prime }=\text{∆}C\;t$$ (4)

where *t′* represents the corrected trajectory that is consistent with the intraoperative alignment. This formulation ensures that the geometric relationship between the screw path and the vertebral anatomy is preserved while adapting to pose changes.

Figure 5 illustrates the effect of the realignment process. When the preoperative trajectory is directly applied to a shifted intraoperative configuration, misalignment is observed. By contrast, the transformed trajectory remains aligned with the vertebral structure after correction.

**Fig. 5.**
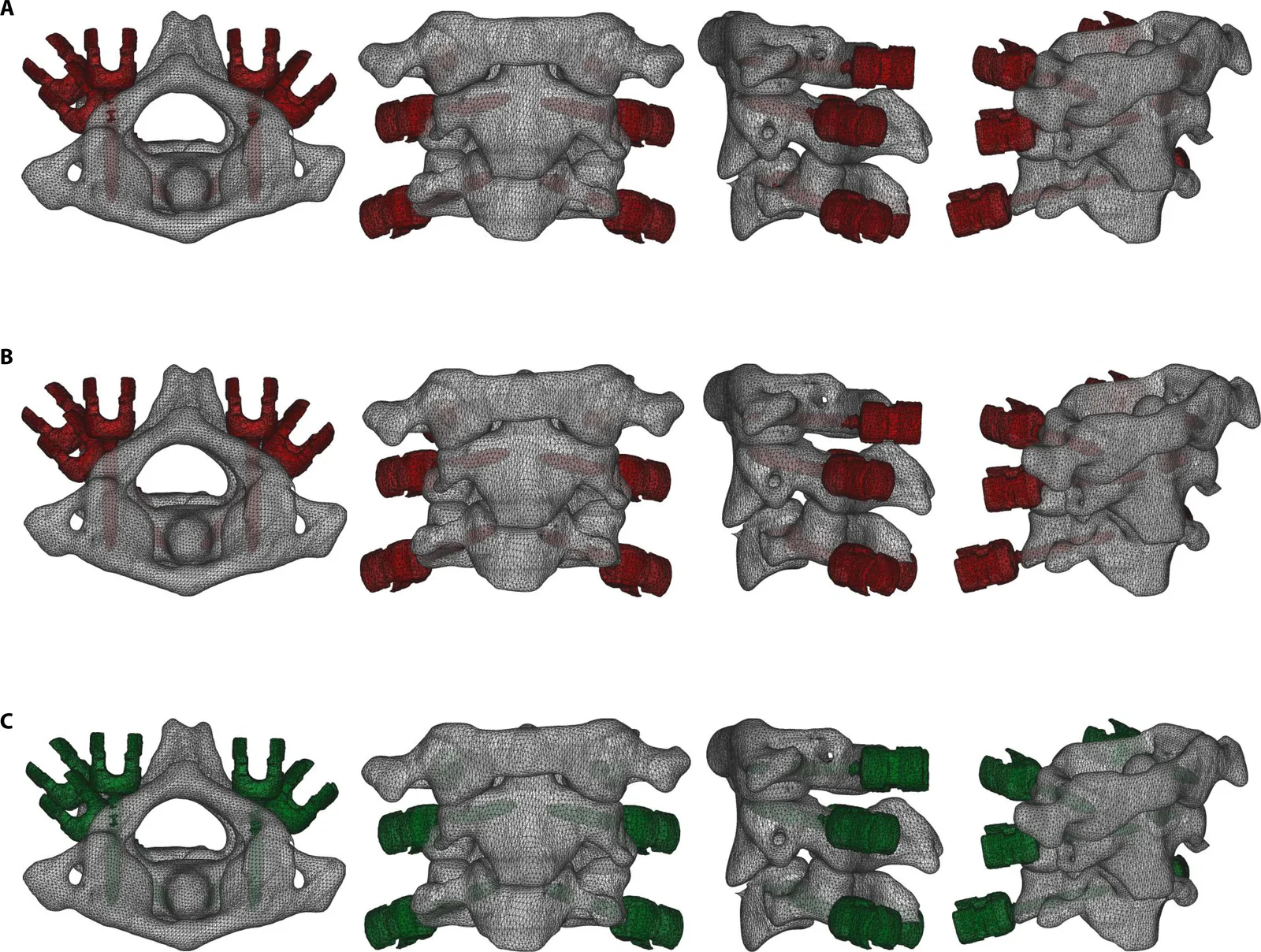
Illustration of trajectory realignment: (A) preoperative configuration with planned trajectory, (B) intraoperative configuration with uncorrected trajectory, and (C) intraoperative configuration after trajectory correction.

The complete workflow, combining pose estimation and trajectory updating, is summarized in Fig. 6. This integrated process enables systematic adjustment of preoperative plans based on intraoperative alignment changes.

**Fig. 6.**
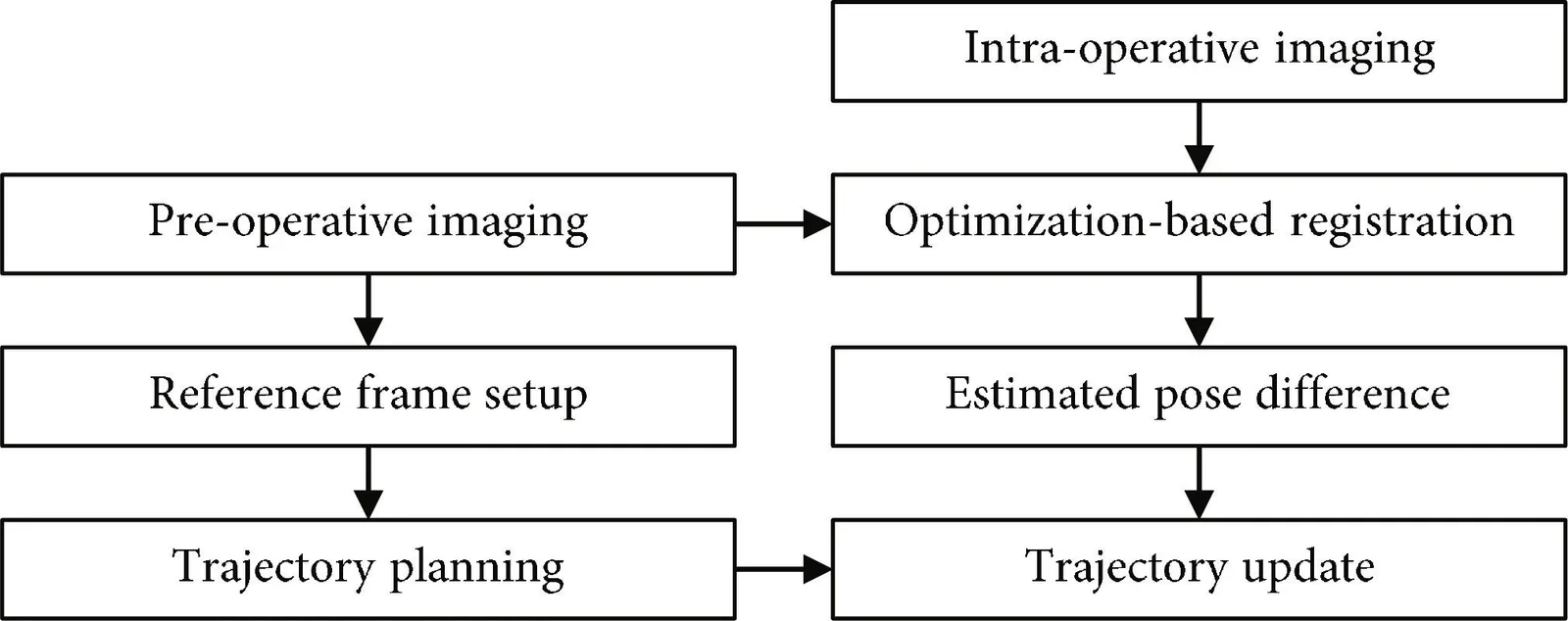
Overall framework for spine image registration and trajectory realignment.

## Computational Experiment and Results

The performance of the proposed realignment algorithm was evaluated using a controlled computational framework designed to simulate intraoperative alignment variations. Patient-specific cervical spine data were obtained from the CTSpine1K dataset [31]. A subset of 9 cases was selected, and analysis was restricted to the upper cervical levels (C1 to C3), resulting in a total of 27 vertebrae and 54 corresponding screw trajectories.

Preoperative planning was performed using 3D reconstructions of the segmented vertebrae. Screw trajectories were defined within the 3D Slicer environment following standard surgical planning principles [52,53]. This step established a reference configuration representing the intended surgical trajectory under ideal anatomical alignment.

To emulate intraoperative conditions, vertebral poses were systematically perturbed using randomly generated parameters constrained by physiological ranges of motion. This approach allowed the simulation of alignment discrepancies arising from posture changes and spinal mobility. While simplified, this setup provides a controlled environment for assessing the ability of the algorithm to recover the underlying alignment.

The proposed registration and realignment pipeline (see Spine Kinematics and Spine Image Registration and Trajectory Realignment sections) were then applied to estimate the transformed vertebral poses and update the corresponding screw trajectories. Algorithm performance was evaluated using both clinically relevant grading criteria and geometric trajectory error metrics [54,55].

### Clinical grading system

Clinical accuracy was assessed using a widely adopted grading scheme based on cortical breach distance, as summarized in Table 2. This classification evaluates the extent to which a screw trajectory deviates outside the cortical boundary, providing a clinically interpretable measure of placement accuracy.

**Table 2.** Grading system for screw insertion accuracy

Grade	Description
0	No cortical breach (contained within vertebral boundaries)
1	Breach less than 2 mm
2	Breach between 2 and 4 mm
3	Breach greater than 4 mm

Although originally developed for thoracolumbar instrumentation, this grading system has been extensively applied in cervical spine studies, as the underlying principle of evaluating cortical violation remains consistent across spinal regions. In this study, grading was performed by visual inspection of reconstructed trajectories in 3D Slicer, allowing direct assessment of screw positioning relative to vertebral boundaries.

### Trajectory difference evaluation

In addition to clinical grading, quantitative geometric analysis was performed to evaluate the agreement between the planned trajectory and the trajectory updated by the proposed algorithm [56]. This analysis provides a detailed assessment of spatial deviations in both position and orientation, which are critical for determining the accuracy of screw placement.

Four complementary metrics were defined, as illustrated in Fig. 7.

1. Entry point deviation: The positional discrepancy at the screw entry site was quantified using the Euclidean distance between the entry point of the reference trajectory and that of the updated trajectory:
  $$D_{ent}=∥P_{1,r}-P_{1,u}∥$$ (5)

where *P*_1*,r*_ and *P*_1*,u*_ denote the entry points of the planned and updated trajectories, respectively.

2. Exit point deviation: Similarly, the deviation at the distal end of the trajectory was computed as:
  $$D_{ext}=∥P_{2,r}-P_{2,u}∥$$ (6)
  where *P*_2*,r*_ and *P*_2*,u*_ represent the exit points of the planned and updated trajectories.
3. Angular deviation: The difference in trajectory orientation was evaluated by calculating the angle between the direction vectors of the planned and updated trajectories:
  $$\theta ={\text{cos}}^{-1}\frac{A\cdot B}{∥A∥∥B∥}$$ (7)

where **A** = *P*_2*,r*_ *− P*_1*,r*_ and **B** = *P*_2*,u*_ *− P*_1*,u*_ are the direction vectors of the reference and updated trajectories, respectively.

4. Minimum distance between trajectories: To assess the overall spatial discrepancy along the entire trajectory path, the shortest distance between the 2 lines was calculated as:
  $$D_{\text{min}}=\frac{∥(P_{1}-P_{2})\cdot \left(u\times v\right)∥}{∥\left(u\times v\right)∥}$$ (8)
  where **r**_1_(*t*) = **P**_1_ + *t***u** and **r**_2_(*s*) = **P**_2_ + *s***v** represent the parametric forms of the planned and updated trajectories, respectively. Here, **u** and **v** are their direction vectors.

**Fig. 7.**
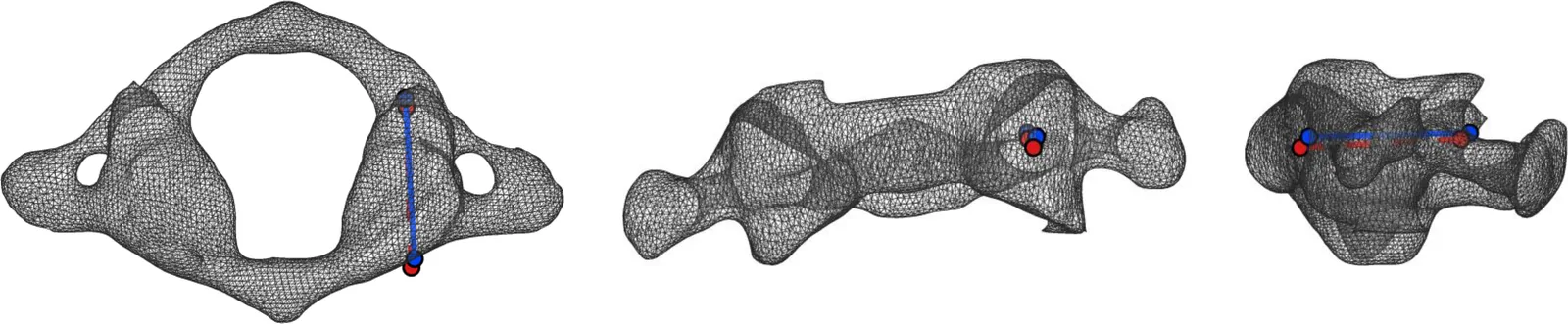
Geometric evaluation of trajectory alignment. Blue lines indicate the planned trajectory, while red lines represent the updated trajectory estimated by the algorithm. The evaluation metrics include entry and exit point deviations, angular difference, and minimum distance between trajectories.

Together, these metrics capture both local (entry/exit point) and global (orientation and spatial alignment) discrepancies between trajectories. This multidimensional evaluation framework enables a comprehensive assessment of the algorithm’s geometric accuracy and complements the clinically oriented grading system described above.

### Experimental results

The quantitative performance of the proposed trajectory realignment algorithm is summarized in Table 3, which reports descriptive statistics for the 4 evaluation metrics. Across all simulated cases, the mean deviation at the entry and exit points was approximately 1.18 and 1.21 mm, respectively. The average angular deviation remained below 1°, while the minimum distance between trajectories was 0.63 mm on average. These values indicate close geometric agreement between the planned and updated trajectories under the simulated conditions.

**Table 3.** Statistical analysis of the algorithm performance based on 4 key parameters in the computational experiment

Parameter	Average	SD	Max	Min
Distance between entry points	1.1793	0.6964	2.3102	0.0094
Distance between exit points	1.2071	0.6852	2.3383	0.0227
Angle difference	0.9701	1.0028	4.1425	0.0084
Minimum distance between lines	0.6320	0.5944	1.9479	0.0041

To provide additional insight into variability, inferential statistics including the standard error of the mean (SEM) and 95% confidence intervals are presented in Table 4. While these intervals demonstrate low sample variability, they should be interpreted within the context of the study’s clustered data structure. Because the 54 evaluated trajectories originate from 9 patient anatomical models, the data points represent repeated intrapatient measures rather than strictly independent observations. Consequently, these metrics reflect algorithmic consistency across the tested anatomies, and broader population generalizability remains to be established across larger cohorts.

**Table 4.** Inferential statistics (SEM and 95% CI) of the algorithm performance based on 4 key parameters in the computational experiment

Parameter	SEM	^CI^low	^CI^high
Distance between entry points	0.0948	0.9892	1.3694
Distance between exit points	0.0932	1.0200	1.3941
Angle difference	0.1365	0.6964	1.2438
Minimum distance between lines	0.0804	0.4698	0.7942

Based on the clinical grading criterion, all trajectories remained within cortical boundaries in the simulated environment, corresponding to grade 0 classification. While this reflects high alignment accuracy under controlled conditions, it is important to note that the absence of imaging noise, soft tissue interference, and other intraoperative complexities may contribute to this ideal outcome.

Figure 8 illustrates the displacement between planned and updated trajectories at both entry and exit points across all samples. Consistent trends can be observed, with relatively small deviations distributed across cases. Figure 9 presents the distribution of angular deviations for individual cervical levels.

**Fig. 8.**
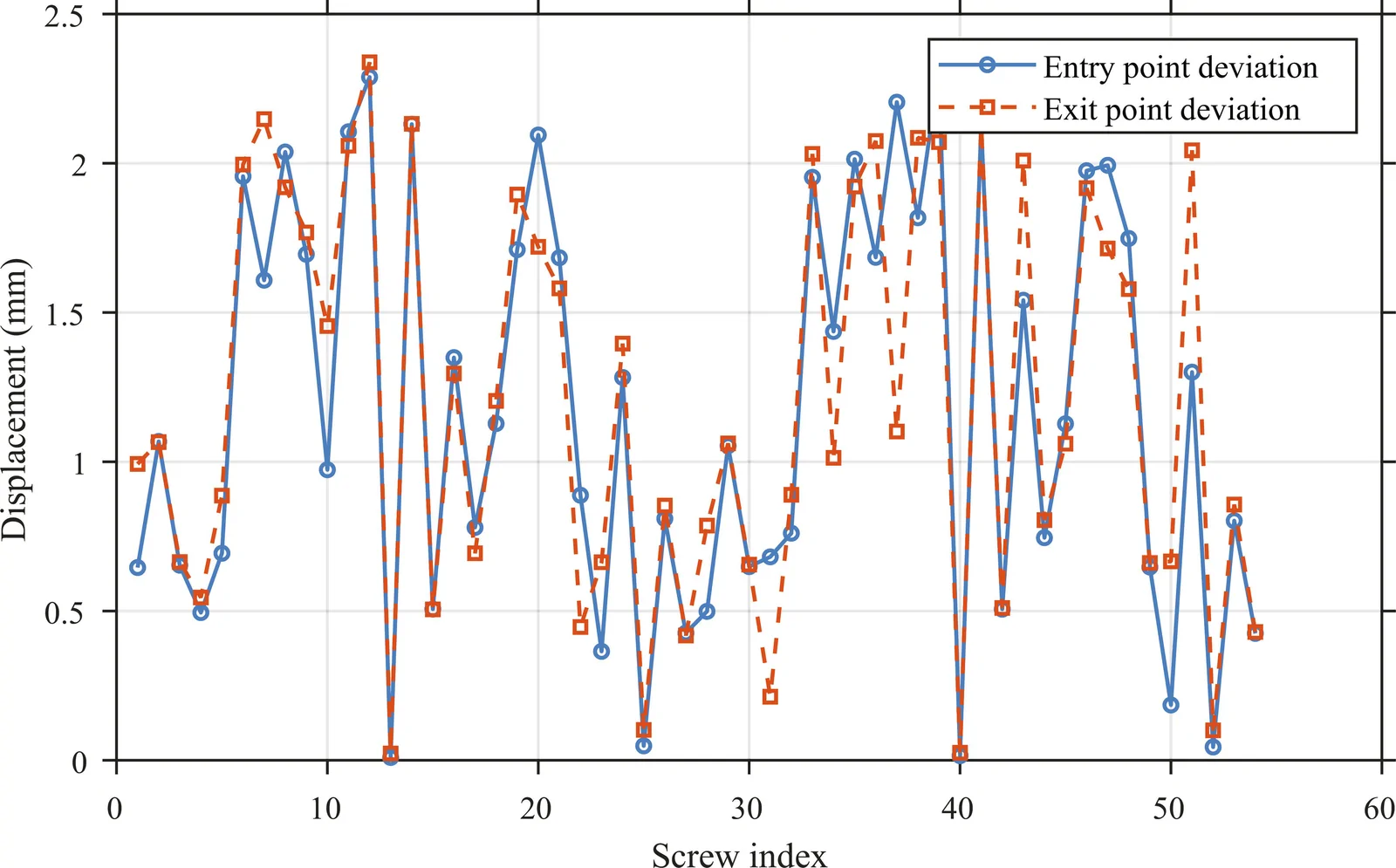
Displacement of entry and exit points between planned and updated trajectories across simulated cases.

**Fig. 9.**
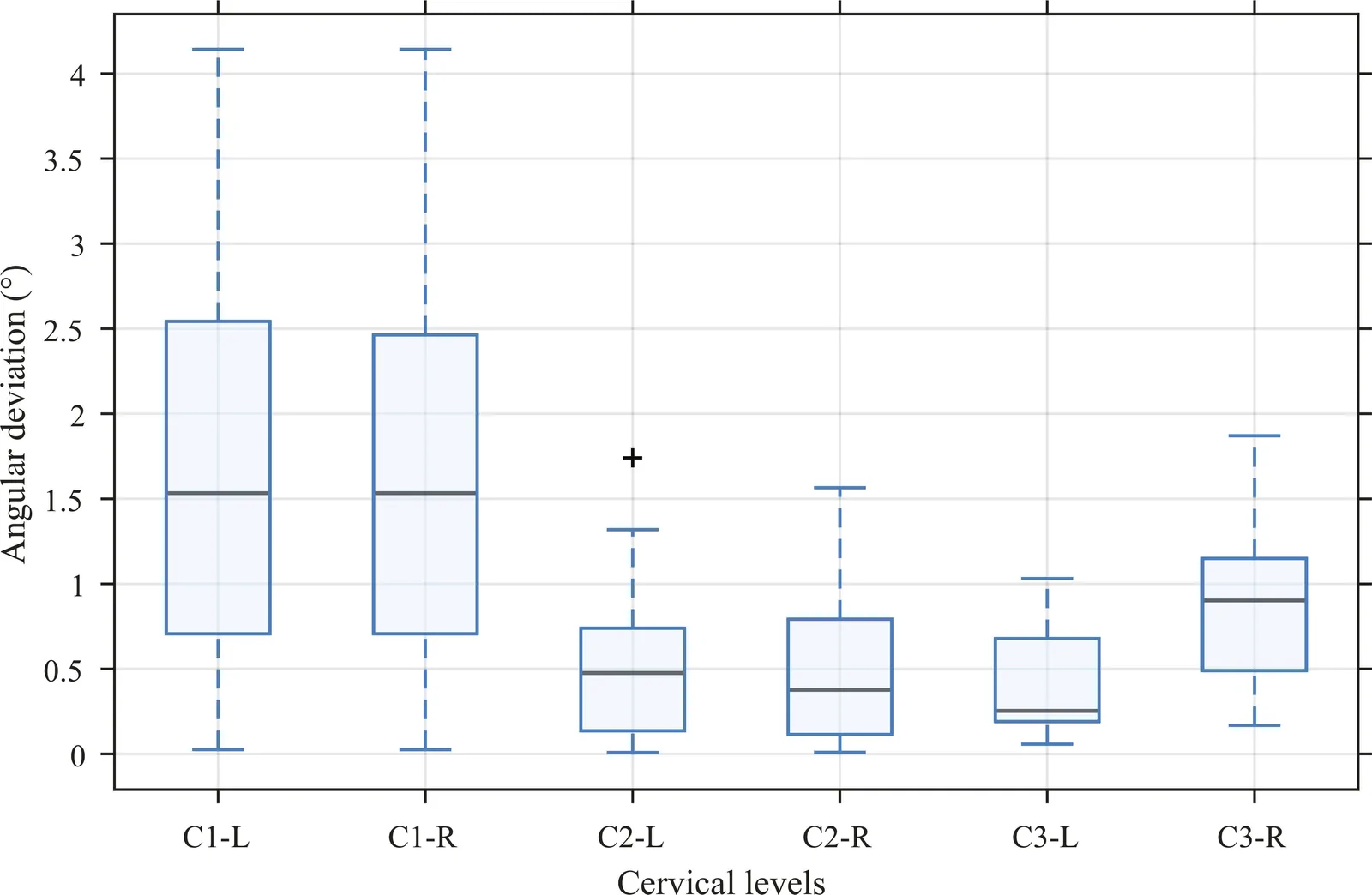
Distribution of angular deviations across cervical levels (C1 to C3) in the computational experiment.

Analysis of angular deviation indicates a decreasing trend from C1 to lower cervical levels. This trend suggests that while the registration of C1 is performed against the full range of random initial pose perturbations, the downstream registration for C2 and C3 benefits from the stabilized reference frame established by the preceding vertebral alignment. In this sequential optimization framework, the kinematic chain propagates constraints that effectively restrict the search space for distal segments, contributing to the observed improvement in consistency at lower levels. However, this phenomenon must be interpreted with caution; while it demonstrates the stability of the hierarchical chain, it implies that the improved consistency at distal levels relies partly on these propagated constraints rather than entirely independent registration accuracy. This underscores the theoretical risk of cumulative bias, wherein persistent inaccuracies in a proximal segment could influence distal alignment estimates, warranting future investigation into global refinement mechanisms.

Overall, the results demonstrate that the proposed method is capable of recovering trajectory alignment with low geometric error in a controlled computational environment. Because this in silico environment excludes soft tissue attenuation, scatter artifacts, fluoroscopic noise, metal artifacts, and surgical instrumentation, these results must be interpreted strictly as an idealized upper-bound estimate of algorithmic feasibility rather than an indicator of clinical readiness.

## Phantom Experiment and Results

To further assess the feasibility of the proposed trajectory realignment algorithm beyond simulation, a controlled physical phantom experiment was conducted using 3D printed cervical vertebrae.

### Phantom design and setup

A modular cervical spine phantom was constructed from CT-based anatomical models derived from the CTSpine1K dataset [31]. The phantom design incorporated a custom mechanical base that allowed controlled rotational adjustment of the C1 and C2 vertebrae while maintaining their centroid positions. This configuration ensured consistency with the reference frame definition used in the kinematic modeling and registration framework, as illustrated in Fig. 10 [52,53].

**Fig. 10.**
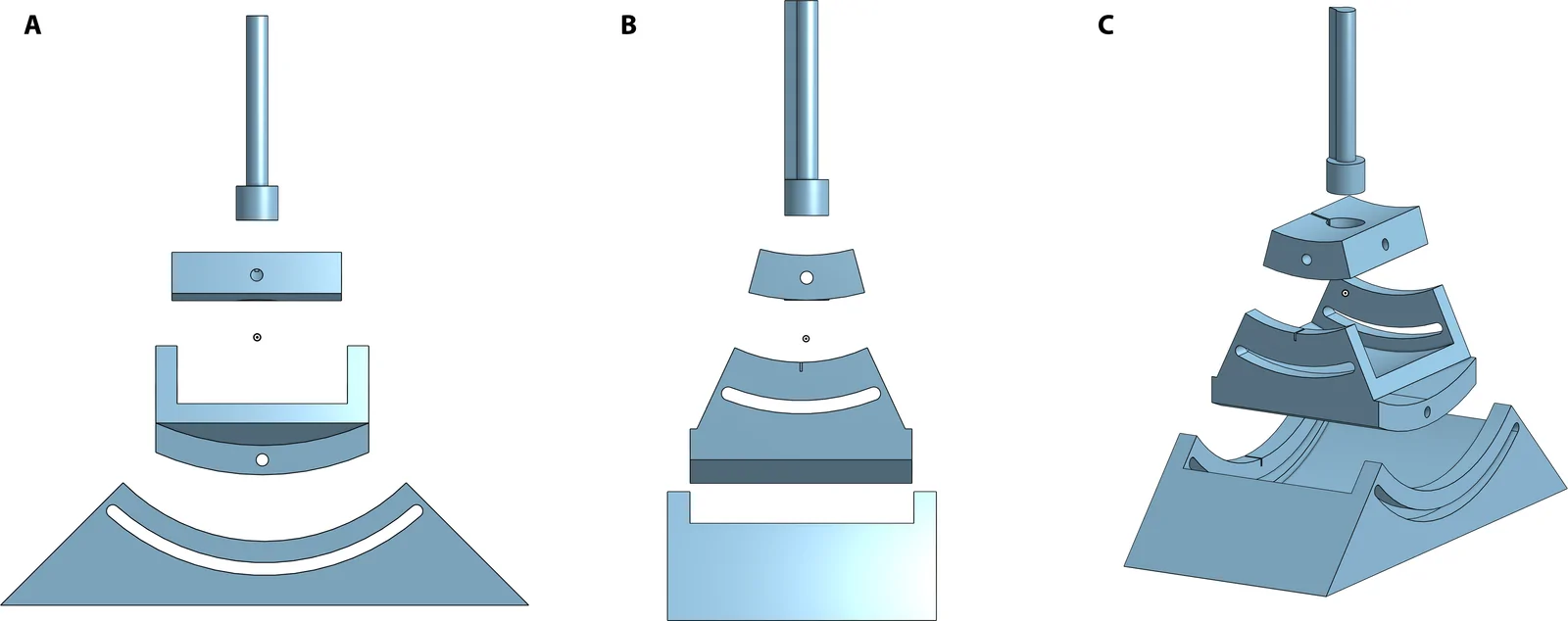
Configurations of the modular cervical spine phantom: (A) front view, (B) lateral view, and (C) oblique view.

This experimental component represents a key extension beyond our prior conference study [27], which was limited to computational validation, by introducing a physical phantom setup to evaluate the algorithm under more realistic and constrained conditions. Crucially, to establish clear boundaries between the 2 experimental phases early on, it should be noted that while the computational simulations evaluated a full 6-degree-of-freedom pose, the phantom experiments were explicitly restricted to a 2-degree-of-freedom optimization consisting solely of rotational parameters.

The experimental protocol was designed to mirror the computational workflow while introducing physical variability. Due to practical constraints, the phantom model included 2 vertebrae (C1 and C2), which were configured into 5 distinct poses within physiologically plausible rotational ranges. This resulted in a total of 10 experimental scenarios. Image acquisition was performed using a calibrated digital camera with fixed intrinsic parameters, as shown in Fig. 11.

**Fig. 11.**
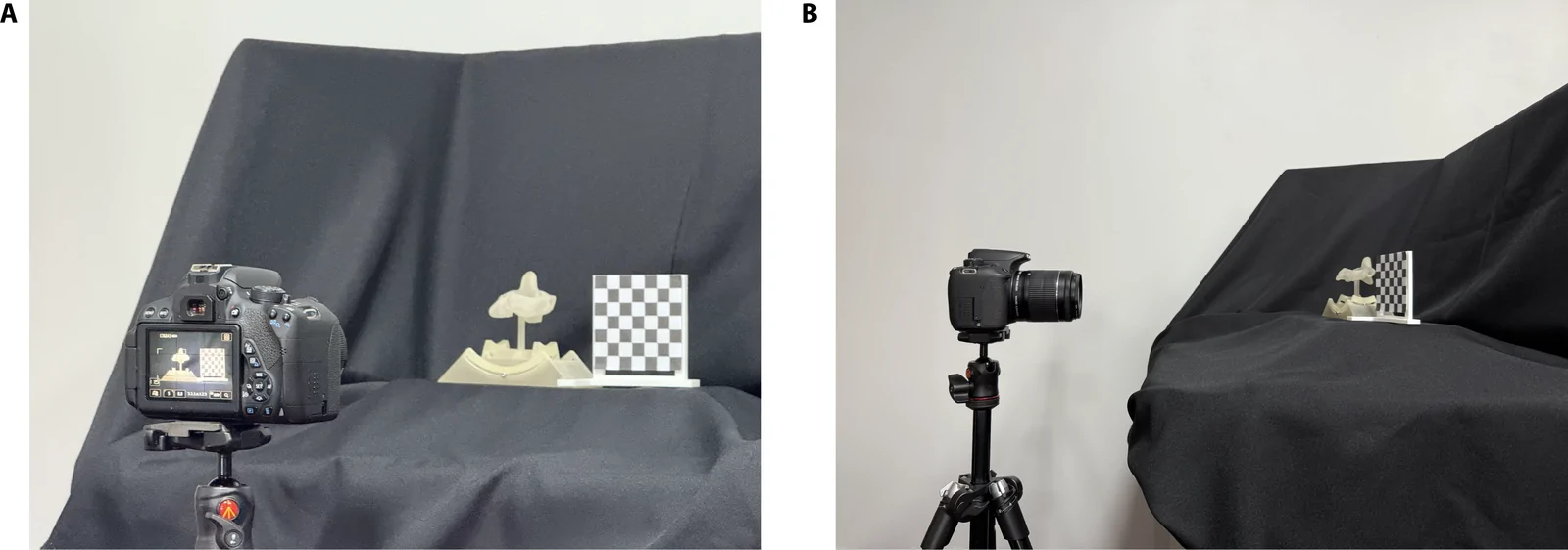
Experimental setup for phantom data acquisition: (A) camera view and (B) lateral view of the phantom.

Prior to analysis, all captured images underwent preprocessing to ensure geometric consistency. Camera lens distortion was corrected using a checkerboard-based calibration procedure, followed by segmentation of the vertebral structures into binary masks. Unlike the grayscale projections used in the computational experiments, the phantom images contained only silhouette information, representing the outer contour of the bone structures. The preprocessing steps are illustrated in Figs. 12 and 13 [57–60].

**Fig. 12.**
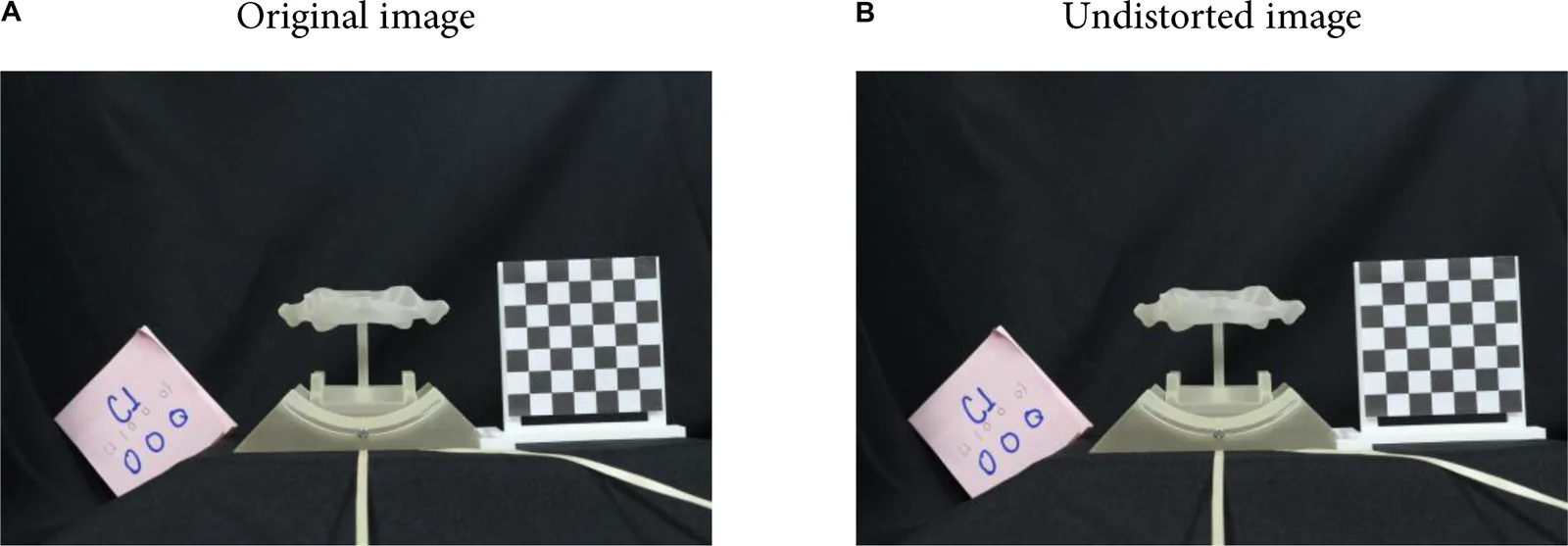
Lens distortion correction using checkerboard calibration. Original image (A) and undistorted image (B).

**Fig. 13.**
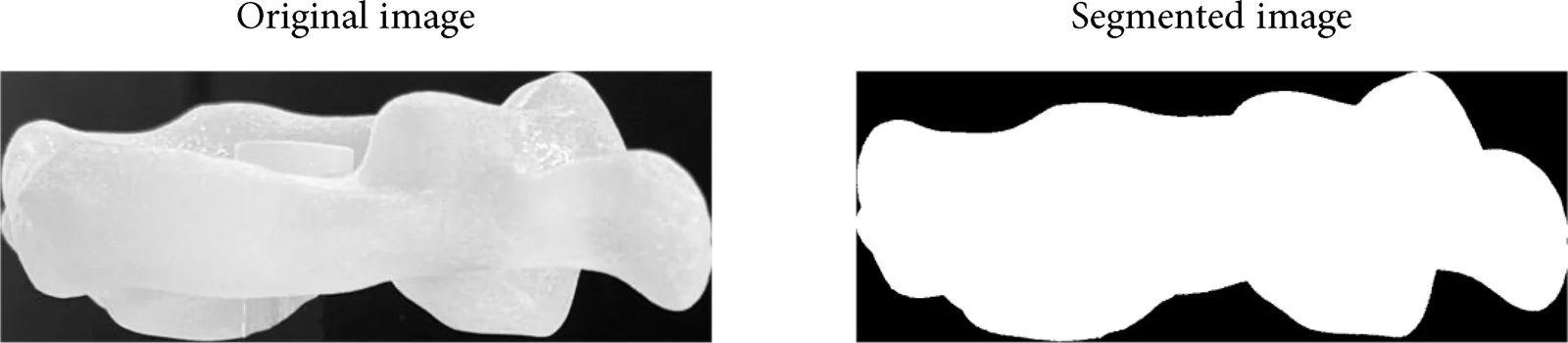
Segmentation of the undistorted image into a binary representation for algorithm input.

Calibration accuracy was evaluated using reprojection error, defined as the Euclidean distance between detected checkerboard corners and their corresponding projections from the estimated camera model [57–59]. The calibration process yielded a mean reprojection error of 0*.*77 *±* 0*.*66 pixels, with a maximum error of 5.01 pixels, indicating acceptable geometric accuracy for subsequent analysis.

Compared to the computational experiment, the phantom setup introduces several simplifying assumptions. First, due to the use of cropped binary images and a single-level configuration, the optimization was restricted to rotational parameters, while translational degrees of freedom were not estimated. Second, the input data lacked grayscale intensity information, which is typically available in fluoroscopic imaging. As a result, only coarse geometric features were used for registration. These simplifications reduce the complexity of the problem and should be considered when interpreting the results.

### Experimental results

The performance of the proposed algorithm in the phantom setting is summarized in Table 5. Across all test scenarios, the mean positional deviation at the entry and exit points was below 1 mm, with average values of 0.77 mm and 0.74 mm, respectively. The mean angular deviation was 2.05°, and the average minimum distance between trajectories was 0.0945 mm, indicating close geometric alignment under the experimental conditions.

**Table 5.** Performance of the algorithm across 4 key parameters in the phantom experiment

Parameter	Average	SD	Max	Min
Distance between entry points (mm)	0.7678	0.5322	1.9305	0.1060
Distance between exit points (mm)	0.7399	0.4762	1.8282	0.0901
Angle difference (degrees)	2.0500	1.4020	4.6812	0.0723
Minimum distance between lines (mm)	0.0945	0.1207	0.4620	0.0093

Inferential statistics, including the SEM and 95% confidence intervals, are presented in Table 6. The observed variability is higher than in the computational experiment, reflecting the influence of physical factors such as mechanical tolerances in the phantom structure and image acquisition variability.

**Table 6.** Inferential statistics (SEM and 95% CI) of the algorithm performance based on 4 key parameters in the phantom experiment

Parameter	SEM	^CI^low	^CI^high
Distance between entry points	0.1190	0.5187	1.0169
Distance between exit points	0.1065	0.5171	0.9628
Angle difference	0.3135	1.3939	2.7062
Minimum distance between lines	0.0270	0.0380	0.1510

All evaluated trajectories remained within the cortical boundaries in the phantom environment, corresponding to grade 0 classification. While this indicates accurate alignment under the experimental setup, the absence of imaging noise, soft tissue attenuation, and fluoroscopic artifacts should be considered when interpreting these results.

Figures 14 and 15 illustrate the distribution of positional and angular deviations across test scenarios. Compared to the computational experiment, the phantom results exhibit slightly increased variability, which is consistent with the introduction of physical uncertainties during image acquisition and model manipulation.

**Fig. 14.**
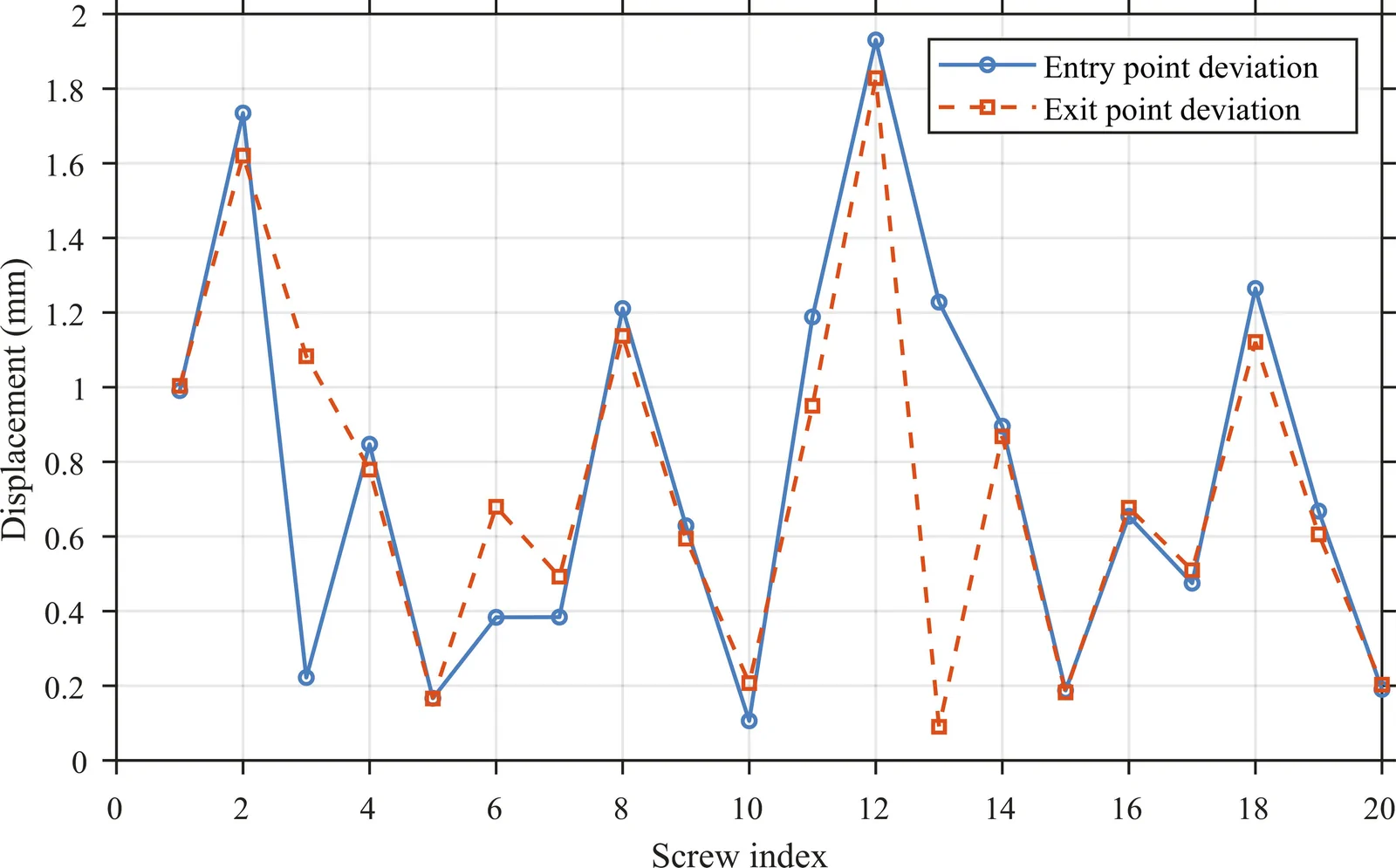
Displacement of entry and exit points between planned and updated trajectories in the phantom experiment.

**Fig. 15.**
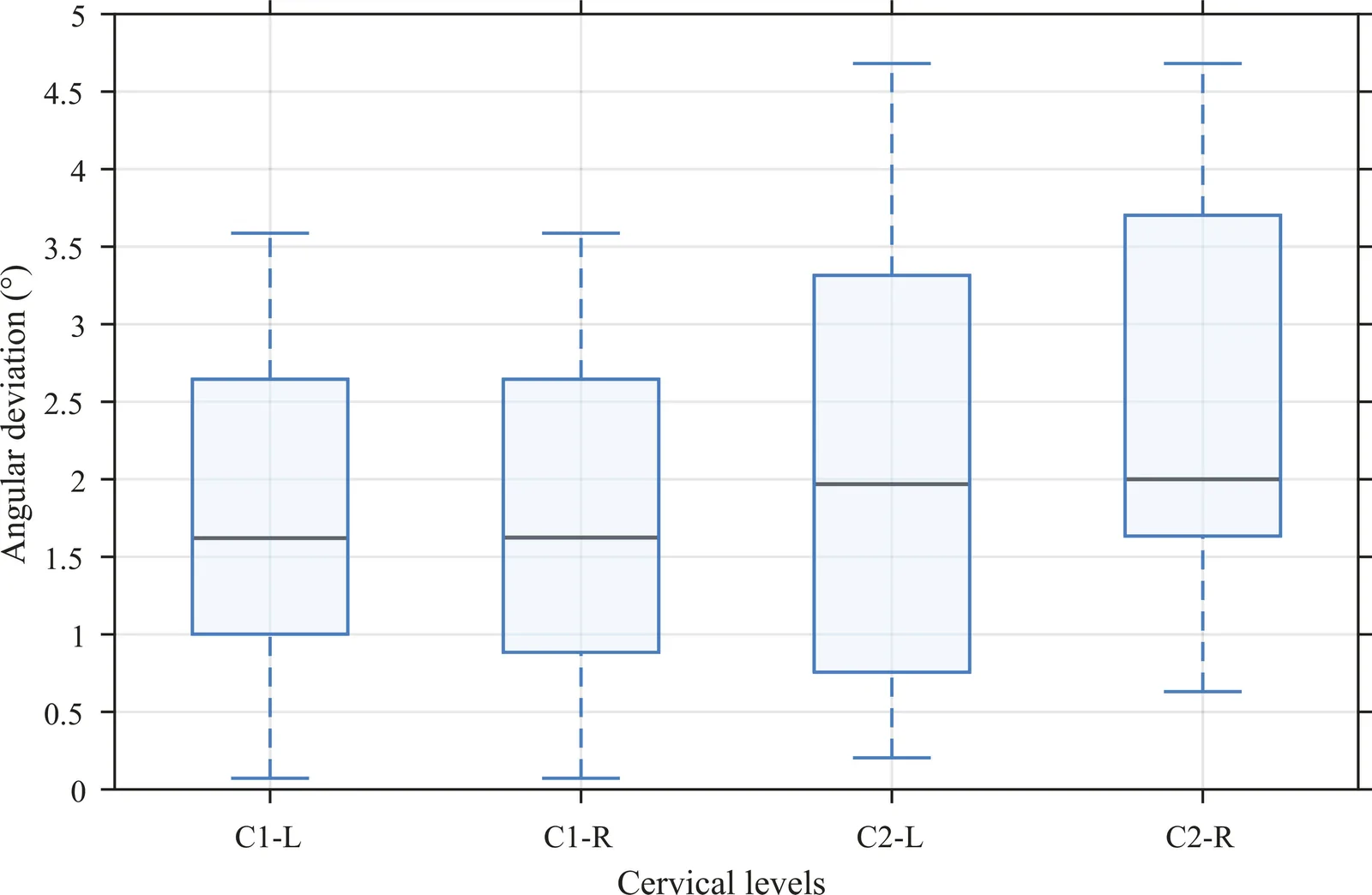
Distribution of angular deviations across phantom test scenarios.

Several limitations of the phantom setup should be noted. First, the use of binary silhouette images restricts the available image information to outer contours, without internal anatomical features. Second, translational degrees of freedom were not included in the optimization, resulting in a reduced parameter space. These simplifications decrease the complexity of the registration problem and may influence the observed accuracy.

Overall, the phantom experiment demonstrates that the proposed method can maintain low geometric error under controlled physical conditions. However, because this setup relied on high-contrast binary silhouette masks, fixed camera geometry, controlled lighting, and rotational-only perturbation, the registration complexity was substantially lower than that of real fluoroscopic spine surgery. Consequently, this physical validation serves strictly as a foundational feasibility model, necessitating substantial future testing under degraded, realistic surgical imaging conditions before clinical translation can be considered.

## Baseline Comparison

To evaluate the influence of the similarity metric on registration performance, a baseline comparison was conducted using 3 commonly applied measures: SAD, normalized cross-correlation (NCC), and mutual information (MI). The experiment was performed on 3 cervical levels from a single patient, resulting in a total of 6 screw trajectories per method. All optimization settings were kept identical across experiments, with only the similarity metric varied within the cost function.

Performance was assessed using the same evaluation protocol as described in the computational experiment. The results are summarized in Tables 7 and 8, including both descriptive statistics and inferential measures.

**Table 7.** Comparison of similarity metrics (SAD, MI, NCC) in the baseline experiment, evaluated on 4 trajectory parameters

Parameter	Method	AVG	SD	Min	Max
Entry distance (mm)	SAD MI NCC	0.7724 2.9519 1.4287	0.3225 1.9572 0.9764	0.4650 0.4206 0.5668	1.1623 4.6145 2.9403
End distance (mm)	SAD MI NCC	0.8482 3.2530 1.2537	0.3153 2.0244 0.6789	0.4731 0.6657 0.5447	1.2214 5.0923 2.3820
Angle difference (°)	SAD MI NCC	0.7768 4.0762 0.9059	0.6549 3.7658 1.0515	0.1718 0.5829 0.0779	1.5988 8.9858 2.2428
Minimum distance (mm)	SAD MI NCC	0.2173 1.6076 0.5609	0.2294 1.8535 0.4524	0.0104 0.0760 0.0230	0.6455 4.0032 1.1816

**Table 8.** Inferential statistics (SEM and 95% CI) of similarity metrics (SAD, MI, NCC) in the baseline experiment

Parameter	Method	SEM	95% CI_low_	95% CI_high_
Entry distance (mm)	SAD MI NCC	0.1317 0.7990 0.3986	0.434 0.898 0.404	1.111 5.006 2.453
End distance (mm)	SAD MI NCC	0.1287 0.8265 0.2772	0.517 1.129 0.541	1.179 5.378 1.966
Angle difference (°)	SAD MI NCC	0.2674 1.5374 0.4293	0.090 0.124 −0.198	1.464 8.028 2.009
Minimum distance (mm)	SAD Mi NCC	0.0937 0.7567 0.1847	−0.024 −0.338 0.086	0.458 3.553 1.036

Statistical analysis using the Kruskal–Wallis test followed by Dunn–Šidák post hoc comparisons did not reveal significant differences among the 3 methods for entry and exit point deviations (*P >* 0*.*05) [61]. For angular deviation, the overall test indicated a marginal effect (*P* = 0*.*048); however, this did not remain significant after correction for multiple comparisons. No statistically significant differences were observed for the minimum distance metric.

Although statistical significance was not established, differences in performance trends were observed. SAD and NCC generally produced lower and more consistent geometric deviations across all parameters, whereas MI exhibited greater variability, as reflected by its larger standard deviations and wider confidence intervals. This behavior may be attributed to the sensitivity of MI to intensity distribution, which can be less stable in the simplified imaging conditions used in this study.

It is important to note that the limited sample size in this experiment restricts the statistical power of the analysis, and the results should therefore be interpreted as exploratory. Within this context, SAD provides a stable and computationally efficient similarity measure, which supports its selection as the primary metric in the proposed registration framework. NCC demonstrated comparable performance and may serve as an alternative in similar settings, while MI may require more complex imaging conditions to achieve consistent results. It should be noted that the present comparison is limited to classical intensity-based similarity metrics and does not include more advanced registration approaches, such as feature-, gradient-, or learning-based methods.

Overall, these findings suggest that simpler intensity-based metrics such as SAD remain effective for the current application, particularly under controlled imaging assumptions. Further evaluation using larger datasets and more realistic intraoperative imaging conditions is necessary to establish definitive conclusions.

## Computational Efficiency

The computational performance of the proposed algorithm was evaluated to assess its feasibility for intraoperative use. All experiments were implemented in MATLAB R2023a and executed on a workstation equipped with an Intel Core i7-13700H CPU (2.4 GHz, 14 cores, 20 threads) and 16 GB RAM, running Microsoft Windows 11 (64-bit). Graphics processing unit (GPU) acceleration was not utilized. Parallel processing was enabled via MATLAB’s Parallel Computing Toolbox, allowing concurrent evaluation of candidate solutions within the pattern search algorithm.

For each optimization, both runtime and the number of cost function evaluations were recorded. On average, convergence was achieved after 1,089.67 function evaluations, corresponding to a mean runtime of 1,206.91 s. The average computation time per cost function evaluation was 111.12 ms.

Regarding granular profiling, note that detailed data separating discrete DRR generation bottlenecks from mathematical optimization time, or tracking iteration counts independently of total function evaluations, were not harvested during this testing phase. This initial work prioritized methodological validation over computational engineering. Architecturally, however, the DRR generation process acts as the primary computational bottleneck, as it requires high-fidelity voxel-based ray-tracing for each projection. Furthermore, since the framework optimizes vertebral levels independently in a sequential hierarchical chain, computational complexity scales linearly with the number of segments processed.

While the algorithm demonstrated stable convergence across all experiments, the current computational performance is not sufficient for real-time intraoperative deployment, where response times are typically expected to be within seconds to a few minutes. The present implementation prioritizes methodological validation rather than execution speed, and therefore does not incorporate optimization strategies for computational efficiency.

Several approaches may be considered to improve performance in future work. These include accelerating DRR generation using GPU-based rendering, adopting multi-resolution or coarse-to-fine optimization strategies to reduce the search space, and implementing more efficient initialization schemes to improve convergence speed. Additionally, transitioning from interpreted MATLAB code to compiled implementations may further reduce runtime.

It should also be noted that the reported runtime is influenced by the current experimental design, including full-resolution image processing and independent optimization of multiple vertebral levels. Algorithmic refinements and hardware acceleration are expected to substantially reduce computation time. Therefore, while the current implementation is not yet suitable for real-time application, the results support the feasibility of the proposed approach as a foundation for future development toward clinically practical systems.

## Conclusions

This study presents a trajectory realignment framework designed to adjust preoperative screw planning according to intraoperative cervical spine alignment. By addressing discrepancies between planned and actual anatomical configurations, the proposed approach aims to improve the consistency of navigation-based trajectory guidance.

### Key achievements

The primary contribution of this work is the integration of forward kinematics, DRRs, and optimization-based image registration into a unified framework for intraoperative trajectory adjustment. While these components have been explored individually in prior studies, their combined application to trajectory realignment provides a structured approach for addressing alignment inconsistencies in preoperative image-based navigation.

Compared with our previous conference study [27], which primarily focused on algorithm development and computational validation, the present work extends the framework through phantom-based experimentation, baseline similarity-metric comparison, and a more comprehensive evaluation of performance and limitations.

The proposed method demonstrated consistent geometric performance in both computational and phantom experiments. In the simulation setting, low deviations were observed across positional and angular metrics, reflecting accurate recovery of alignment under controlled conditions. The phantom experiment further evaluated the method in a physical environment, where slightly increased variability was observed, but overall alignment accuracy was maintained.

These findings indicate that the proposed framework is capable of adapting planned trajectories to changes in spinal alignment within simplified experimental settings. However, it is important to note that the current validation does not fully represent the complexity of intraoperative imaging, including factors such as fluoroscopic noise, soft tissue interference, and full 6-degree-of-freedom motion.

Overall, this study establishes a proof-of-concept for trajectory realignment using image-based registration and kinematic modeling. Further investigation using clinically realistic imaging modalities and larger datasets is required to assess its applicability in surgical practice.

### Limitations

Several limitations of this study should be acknowledged. First, the DRR generation employed a simplified imaging model with uniform attenuation properties, without accounting for factors such as heterogeneous bone density, soft tissue contributions, scatter, or imaging noise. Crucially, the algorithm was not subjected to perturbation testing against synthetic degradations, including additive Gaussian noise, partial vertebral occlusion, projection angle variations, or segmentation inaccuracies. Consequently, algorithmic robustness to degraded imaging conditions remains unknown, and the highly favorable results observed in this study must be interpreted strictly as an estimate of upper bound performance achieved within a nearly ideal environment. Second, the current implementation does not incorporate computational optimization for real-time performance, resulting in runtimes that are not yet compatible with intraoperative use. Third, the phantom experiment was limited to 2 vertebral levels and excluded surrounding soft tissues, with image acquisition performed using a standard camera rather than fluoroscopic imaging. These conditions simplify the registration problem and may not fully reflect the complexity of clinical scenarios. In addition, the evaluation was conducted on a limited dataset derived from a single source, and no validation has been performed in cadaveric or *in vivo* settings. These factors should be considered when interpreting the reported performance.

### Recommendations for future study

Future work should focus on improving both the realism and efficiency of the proposed framework. From an imaging perspective, more advanced DRR models incorporating heterogeneous tissue properties, noise modeling, and fluoroscopic characteristics would provide a more realistic evaluation environment. From a computational standpoint, reducing runtime will be essential for clinical applicability. Potential strategies include GPU-accelerated DRR generation, multi-resolution or coarse-to-fine optimization schemes, and more efficient initialization techniques to reduce the number of required iterations. Experimental validation should also be extended to more complex and clinically relevant settings. This includes multi-level cervical spine models with soft tissue representation, as well as evaluation using real intraoperative fluoroscopic images. Ultimately, validation in cadaveric models and integration with existing navigation or robotic systems will be necessary to assess the practical utility of the approach.

In summary, this study demonstrates that kinematic modeling, DRR-based image generation, and optimization-based registration can be integrated into a unified framework for trajectory realignment. Consistently viewed as a methodological feasibility study conducted under controlled imaging conditions, this work demonstrates mathematical stability in near-ideal environments. However, before clinical translation or integration into surgical navigation workflows can be considered, the approach requires substantial future validation to account for soft tissue scatter, fluoroscopic noise, metal artifacts, and full 6-degree-of-freedom physical perturbations.

## Data Availability

The data supporting the findings of this study are available from the corresponding author upon reasonable request.
